# Family Socioeconomic Status and Attention Deficit/Hyperactivity Disorder in Preschool Children: The Mediating Role of Executive Function

**DOI:** 10.3390/ijerph191811608

**Published:** 2022-09-15

**Authors:** Liheng Fan, Wenjing Qing, Yinling Wang, Meichen Zhan

**Affiliations:** 1 Institute of Psychology and Behavior, Henan University, Kaifeng 475004, China; 2 Department of Education, Henan University, Kaifeng 475004, China; 3 School of Mathematics and Statistics, Pingdingshan University, Pingdingshan 467000, China

**Keywords:** preschool children, family socioeconomic status, attention deficit/hyperactivity disorder, executive function

## Abstract

This study aimed to explore the relationship between family socioeconomic status (SES) and attention deficit/hyperactivity disorder (ADHD) symptoms in preschool children and the mediating role of executive function (EF). A total of 361 parents of preschool children were surveyed using the self-reported Family Situation Questionnaire, the Child Executive Functioning Inventory, and the Child Strengths and Difficulties Questionnaire. The results revealed that (1) there were significant pairwise correlations between SES, EF and its dimensions, and ADHD, except for a non-significant correlation between SES and regulation ability; (2) after controlling for preschool children’s age and sex, SES directly predicted preschoolers’ ADHD and EF partially mediated the association between SES and ADHD; and (3) among the EF dimensions, working memory and inhibitory ability significantly mediated the association between SES and ADHD, whereas the mediating effect of regulatory ability was not significant. These results suggest that SES can affect the ADHD of preschoolers both directly and through EF, especially through working memory and inhibitory ability. This supports the family stress model and family investment model of the relationship between SES and the development of children to some extent, and provides a reference for the early prevention of ADHD in children with low SES.

## 1. Introduction

Attention deficit/hyperactivity disorder (ADHD) is a disorder of inattention or short attention span, hyperactivity, and impulsivity that is inappropriate for a person’s age and developmental level and is often accompanied by learning difficulties, conduct disorders, and maladjustment [1]. It typically develops during the preschool years, is mostly detected during school age, can persist into adolescence and adulthood [2], and is a common category of cognitive-psychological disorders among childhood. Studies have found that ADHD negatively affects all aspects of preschool children’s development (e.g., learning, behavior, family, peer relationships, cognitive development, and emotional well-being), and these effects may persist into adulthood [3]. Therefore, attention to ADHD in the preschool stage is important for their lifelong development.

### 1.1. Socioeconomic Status and ADHD in Preschool Children

Ecosystem theory suggests that the family is an important microsystem that plays a vital role in children’s physical and mental development [4]. Among the family factors, socioeconomic status (SES) is a composite reflection of material and intangible resources and position in society that represents the family’s economic, human, and educational capital; it is also an important indicator of an individual’s objective economic status. The family stress model argues that family SES affects the members’ interactions and functioning, and low SES places children in a developmentally high-risk situation, making them prone to reduced self-adaptation and problem behaviors [5]. Low family income is a risk factor for psychopathology and dysfunction in children, and children from families with low SES exhibit more ADHD symptoms than those from families with high SES [6]. Based on this, the following hypothesis is proposed:

**H1.**  *Preschool children from low SES families will exhibit a higher incidence of ADHD.*

### 1.2. The Mediating Role of Executive Function between SES and ADHD

In addition to the direct influence of SES on the children’s ADHD, some researchers have focused on the indirect mechanisms in recent years. For example, it has been found that parental involvement and family conflict play mediating roles between SES and the preschool children’s ADHD [7]. However, in addition to these exogenous factors, executive function (EF) has received attention from researchers as an endogenous factor influencing ADHD in children [8]. EF refers to an individual’s ability to use thinking skills to achieve goals, develop problem-solving capability, and monitor and adjust behavior; it is a higher-level processing activity of the brain through which individuals exercise conscious control over their thoughts and actions. EF is closely related to ADHD. In his model, Barkley [9] argues that EF deficits are a central cause of ADHD because impaired EF causes disorders in the self-control and goal behavior in ADHD patients. Several longitudinal studies have found that impaired EF in preschool children predicts the subsequent emergence and development of ADHD [10,11]. Relevant studies on brain localization have also found that ADHD primarily involves the prefrontal and parietal cortices, cerebellum, and basal ganglia, and EF is an important function associated with all of these brain regions. Moreover, SES can influence the development of EF in children. The family investment model suggests that cognitive stimulation in the family environment is a key factor in the neurocognitive development of children [12]. In this model, parents from lower SES families invest fewer resources in providing their children with cognitively stimulating learning materials and experiences (e.g., books or museum visits), hindering the development of EF. Studies have also found that children with lower SES show poorer performance on EF tasks [13,14,15,16]. Therefore, the following hypothesis is suggested:

**H2.**  *EF has a significant mediating role between SES and preschool children’s ADHD.*

### 1.3. The Role of EF Components in the Relationship between SES and ADHD

The extant literature has mostly studied EF as a whole. However, an increasing number of studies including in cognitive neuroscience have found that different components of EF have different developmental trajectories and prefrontal cortex localizations [17]; therefore, each component should be considered as an independent variable when studying EF. Do the three EF dimensions of working memory, regulation, and inhibition also play a mediating role between SES and ADHD? The extant literature may provide some insights. First, working memory refers to the process of retaining information in consciousness to guide subsequent behavior, and is primarily the ability to store and process information in the mind. SES has been found to significantly and negatively predict the working memory capacity of children [18]. Furthermore, poor working memory is one of the main cognitive deficits in children with ADHD [19]. Thus, working memory may mediate the relationship between family SES and preschool children’s ADHD.

Second, inhibition refers to the ability to consciously control autonomous or dominant responses. This includes the inhibitory control of behaviors that do not meet current needs or are inappropriate, primarily as a process of delaying or withholding drastic responses and making appropriate choices and reactions. Some studies have shown a significant correlation between SES and young children’s inhibitory abilities [18], and Hollister measured the inhibition of 4–5-year-old children using a modified Stroop task [20]. The results showed that participants with low SES had more difficulty using inhibition to resolve conflicts compared to participants with high SES. Furthermore, Wen and Chen showed that inhibition is a core deficit in young children with ADHD [21]. From the above evidence, it can be speculated that inhibition may mediate the relationship between SES and preschool children’s ADHD.

Finally, regulation is the ability to reasonably regulate arousal to achieve a goal and to control the behavioral expression of emotions in a socially acceptable manner, which requires young children to regulate their behavior, attention, arousal, and emotional expression, often in relation to motivation. It has been shown that parental education levels and family income are associated with the self-regulation of children, and that children with higher SES show higher personal behavioral regulation compared to children with lower SES [22]. Additionally, lower regulation is another cognitive deficit seen in children with ADHD [23]. Accordingly, regulation may mediate the relationship between SES and ADHD in preschool children.

**Figure 1:**
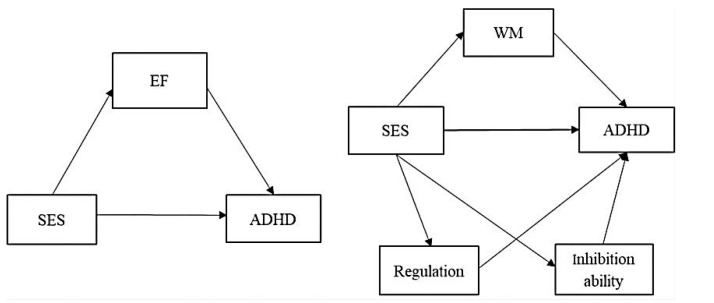
Model of the mediating role of preschool children’s EF and its dimensions between SES and ADHD. SES—socioeconomic status; EF—executive function; ADHD—attention deficit/hyperactivity disorder; WM—working memory. The same applies to the tables in this paper.

Based on the above analysis of the three dimensions of EF, this study proposes H3: There are multiple significant mediating effects of the three components of EF between family SES and ADHD in preschool children (see Figure 1). Specifically,

**H3a.**  *Working memory has a significant mediating effect between SES and ADHD.*

**H3b.**  *Inhibition has a significant mediating effect between SES and ADHD.*

**H3c.**  *Regulation has a significant mediating effect between SES and ADHD.*

## 2. Method

### 2.1. Participants

This study used a convenience sampling method to select young children from small, middle, and large classes in a kindergarten in Kaifeng, China. After obtaining consent from the kindergarten and parents, a questionnaire was administered to the parents of the children on a voluntary basis. After eliminating the invalid data, 361 valid responses were ultimately obtained including 109 (30.2%, 4.42 ± 0.24 years) from the small class, 123 (34.1%, 5.32 ± 0.27 years) from the middle class, and 129 (35.7%, 6.34 ± 0.26 years) from the large class. The distribution of other characteristics on the participants is shown in Table 1. The study protocol followed the APA ethical guidelines and was approved by the participants and the Institutional Review Board of Henan University. 

### 2.2. Measures

#### 2.2.1. Family SES 

Family income and parental education levels were used as assessment indicators. Monthly family income was measured on a 1–5 scale. The father’s and mother’s education levels were measured using one question each on a 1–5 scale. The assessment indices were calculated by first standardizing the scores of family income and parental education levels and then summing the standard scores, with higher scores indicating higher SES.

#### 2.2.2. The Childhood Executive Functioning Inventory (Parent Version)

The Childhood Executive Functioning Inventory was developed by Thorell and Nyberg [24] and translated into Chinese by Wei et al. [25]. In this study, the EF of young children was rated by their primary caregivers. The scale consists of 24 questions and contains three factors (working memory, regulation, and inhibition) that are measured on a scale of 1–5, with higher scores associated with more EF problems. Cronbach’s alpha coefficient for this scale was 0.90, and Cronbach’s alpha coefficients for working memory, regulatory ability, and inhibitory ability dimensions were 0.87, 0.73, and 0.68, respectively.

#### 2.2.3. ADHD

The instrument selected to measure ADHD in children was the hyperactivity subscale of the Strengths and Difficulties Questionnaire, a 5-item scale with a 0–2 three-level subscale, with higher scores indicating more severe ADHD problems in children. This scale has the advantage of being rapid and efficient for screening preschool children for ADHD [26], and Cronbach’s alpha coefficient for this dimension was 0.77.

### 2.3. Procedure

The questionnaire was administered by a highly trained psychology graduate student, and the parents were asked to fill out the questionnaire in the classroom after school; the questionnaire was collected after the parents had completed it. 

### 2.4. Analytic Strategy

The data analysis comprised of four steps: (1) The common method bias of the questionnaire data was tested using factor analysis; (2) the characteristics and relationships of the variables were analyzed using descriptive statistics and correlation analysis; (3) the mediating effects of EF were analyzed using Hayes and Preacher’s [27] bias-corrected percentile bootstrap method (repeated sampling 5000 times with 95% confidence intervals); and (4) the multiple mediating effects of the three components of EF (i.e., working memory, inhibition, and conditioning) were analyzed using the same method as for the mediating effects of EF. Statistical significance was defined as *p* < 0.05.

## 3. Results

### 3.1. Common Method Bias

Since all questionnaire data in this study were obtained from parental self-reports, a Harman one-way test was conducted to control for common method bias effects, and the results showed that there were 15 factors with an eigenvalue greater than 1. The explanatory value of the first common factor was 18.43%, which was less than the threshold (40%), indicating that common method bias was not significant in this study.

### 3.2. Descriptive Statistics and Correlation Analysis

Table 2 depicts the means (*M*) and standard deviations (*SD*) of the preschool children’s SES, EF and its components, and ADHD, and the inter-correlation coefficients. The results revealed bivariate correlations between all variables except for family SES, which was not correlated with the preschool children’s regulation and EF. Each dimension had correlations with ADHD. Sex and age need to be used as control variables for further statistical analyses.

### 3.3. Mediating Effects of EF between SES and ADHD

Table 3 shows that the direct effect of SES on the preschool children’s ADHD was significant after controlling for sex and age. Furthermore, EF partially mediated the effect between SES and ADHD.

### 3.4. Multiple Mediation Effects for Each Dimension of EF between SES and ADHD

Table 4 and Table 5 show that after controlling for sex and age, the mediating effects of working memory and inhibition were significant between SES and ADHD, whereas the mediating effect of regulation was not significant.

A further test for the differences in the mediating effect values was conducted for working memory and inhibition, which was found to be not significant. Thus, SES is mainly associated with preschool children’s ADHD through working memory and inhibitory ability.

## 4. Discussion

### 4.1. Association of SES with EF and ADHD in Preschool Children

This study revealed that the SES of preschool children had a significant negative predictive effect on their ADHD, hence H1 was confirmed; that is, children with lower SES are more likely to display ADHD symptoms. This further validates the family stress model, which states that low SES increases problem behaviors in young children [5,28]. In addition, EF was found to mediate the relationship between SES and ADHD in preschool children, so H2 was confirmed; that is, in addition to directly affecting ADHD, SES also affected ADHD indirectly by influencing the EF of preschool children. Specifically, children with high SES had fewer problems with impaired EF and a lower risk of developing ADHD. The reason for this phenomenon is, according to the family investment model, that parents with high SES may have more resources and energy to invest in their preschool children’s educational activities such as providing learning materials and experiences that promote their cognitive development, thus positively influencing their children’s EF [12]. Moreover, preschool children with high family SES have a more moderated rate and longer duration of age-related reduction in frontal cortex thickness [29], which can have a positive impact on cognitive ability [30]. Thus, a good development of cognitive abilities such as EF will further reduce the occurrence of ADHD in preschool children.

### 4.2. Multiple Mediating Roles of the EF Dimensions between SES and ADHD in Preschool Children

In addition to exploring the mediating role of overall EF between SES and preschool children’s ADHD, this study further examined the multiple mediating roles of the EF dimensions between SES and ADHD. Based on previous literature, this study hypothesized that all dimensions of EF may play a mediating role in the influence of SES on preschool children’s ADHD. However, this study revealed that only working memory and inhibition partially mediated the relationship between SES and preschool children’s ADHD. Therefore, H3a and H3b were confirmed; that is, young children with low SES are more likely to exhibit poorer inhibition and working memory, which, in turn, increases their risk of ADHD. As previously mentioned, this result suggests that, on one hand, low SES families directly affect the development of the brain structure and function in young children by providing them with too few resources and too little interaction [31], leading to the delayed development of inhibition and working memory [32] and, therefore, ADHD [11]. On the other hand, it has been suggested that working memory and inhibition are closely related and are associated with the cognitive load of EF, which Wolfe and Bell refer to as working memory inhibition capacity [33]. The consistency in the role of working memory and inhibition between SES and ADHD found in the present study may also indirectly suggest a close relationship between the two in the structure of EF in young children, and it is likely that in early childhood, working memory and inhibition have not yet reached a stage of differentiation. This result also reminds us that when considering the impact of EF in early childhood, working memory and inhibition can be treated as a single component.

The mediating effect of regulation between SES and preschool children’s ADHD was not significant, so H3c was not confirmed. From the results, it is clear that there was a positive association between regulation problems and ADHD, but the relationship with SES was not significant. Although this may reflect that regulation ability is not a mediating variable between SES and ADHD in young children, it may also be due to the fact that the families selected for this study were from urban areas and were mostly intermediate and higher in SES distribution. Yu et al. found that family poverty hindered the development of the children’s regulation ability [16]. This needs to be supplemented with data on low SES to further validate the explanation.

In summary, this study reveals that the mediating role of the dimensions of EF between SES and preschool children’s ADHD is not consistent, which, to some extent, corroborates the need to examine the three dimensions separately. It also indicates that in studies of EF, it is important to focus on both the role of overall EF and the unique roles and functions of the three dimensions of EF. Moreover, this study revealed that preschool children with low SES may have poorer performance in working memory and inhibition, which may lead to increased severity of ADHD. Therefore, working memory and inhibition training in preschool children with low SES should be focused on when conducting interventions to reduce their risk of ADHD.

### 4.3. Limitations

This study had the following limitations. First, the EF and ADHD of preschool children in this study were assessed by interviewing the parents, reflecting the stable responses of children based on their parents’ observations over a long period of time; however, despite their ecological validity, parents lacked information on immediate performance in cognitive tasks obtained through experimental methods or clinical diagnosis. Therefore, future studies should combine parental assessment and experimental methods to facilitate a balance between the ecological and objective aspects of the study. Second, this study only used a cross-sectional design to build the mediational model; although this method can provide valuable results, it cannot account for the causal relationships among the variables. Thus, future studies should apply a follow-up or experimental design to further validate the causal relationships. Third, the measure of family SES combined family income and parental education levels into one indicator. Vrantsidis et al. argue that SES has a multidimensional structure, and its components may have different effects on EF [32]. Therefore, future studies should consider the economic income, occupation, and education dimensions of SES independently in measuring impact. Fourth, the present study focused only on SES as a family factor and did not investigate the individual characteristics at birth (e.g., weight, prematurity) and maternal behaviors (e.g., alcohol consumption, depression) that may influence ADHD, and in the future, consideration needs to be given as to whether these factors, together with SES, may influence ADHD in young children.

## 5. Conclusions

Young children from families with low SES are more likely to exhibit ADHD symptoms, and EF appears to play a mediating role in the relationship between SES and ADHD. Low SES is associated with impaired inhibitory control and working memory capacities in the EF of young children, which in turn is associated with the emergence of ADHD in some children.

## Figures and Tables

**Table 1:** The sample characteristics.

	*n*	Percentage (%)
Gender (female)	159	44.0
One-child family (yes)	185	51.2
Family income		
Lower than 3000 Yuan	9	2.5
3000 Yuan to 5000 Yuan	59	16.3
5000 Yuan to 7000 Yuan	127	35.2
7000 Yuan to 9000 Yuan	79	21.9
Higher than 9000 Yuan	87	24.1
Parental education levels (mother/father)		
Middle school and below	8/10	2.2/2.8
High school	50/51	13.9/14.1
Junior college	111/117	30.7/32.4
Bachelor’s degree	174/158	48.2/43.8
Graduate degree	18/25	5.0/6.9

**Table 2:** The descriptive statistics results and correlation analysis between variables.

	1	2	3	4	5	6	7	8
1. Sex	1							
2. Age	0.040	1						
3. Working memory	−0.03	−0.11 *	1					
4. Regulation	−0.09	−0.10 *	0.63 ***	1				
5. Inhibition	−0.02	−0.07	0.54 ***	0.63 ***	1			
6. Execution function	−0.09	−0.12 *	0.92 ***	0.83 ***	0.78 ***	1		
7. ADHD	−0.11 *	0.02	0.44 ***	0.45 ***	0.48 ***	0.53 ***	1	
8. SES	−0.08	−0.04	−0.16 **	−0.08	−0.15 **	−0.16 **	−0.20 ***	1
*M*	0.44	5.41	30.94	15.23	17.99	64.16	3.93	0.00
*SD*	0.497	0.82	6.80	3.23	3.40	11.60	2.25	2.14

Notes: Sex: male = 0, female = 1; * *p* < 0.05; ** *p* < 0.01; *** *p* < 0.001, The same applies to all the tables below.

**Table 3:** The regression analysis of SES and overall EF on ADHD in preschool children.

Regression Equation		Overall Fit Index			Significance of Regression Coefficients	
Outcome Variables	Predictor Variables	*R*	*R^2^*	*F*	β	*t*
EF	Sex	0.21	0.04	4.49 **	−0.07	−0.72
	Age				−0.12	−2.32 *
	SES				−0.08	−3.09 **
ADHD	Sex	0.55	0.31	33.70 ***	−0.22	−2.44 *
	Age				0.07	1.69
	SES				−0.06	−2.87 **
	EF				0.51	9.97 ***

**Table 4:** The regression analysis of the SES and EF dimensions on ADHD in preschool children.

Regression Equation		Overall Fit Index			Significance of Regression Coefficients	
Outcome Variables	Predictor Variables	*R*	*R* ^2^	*F*	β	*t*
Working memory	Sex	0.20	0.04	4.58 **	−0.10	−0.91
	Age				−0.12	−2.35 *
	SES				−0.08	−3.09 **
Regulation	Sex	0.14	0.02	1.88	0.01	0.10
	Age				−0.11	−1.92 *
	SES				−0.04	−1.63
Inhibition	Sex	0.17	0.03	3.33 *	−0.08	−0.74
	Age				−0.08	−1.46
	SES				−0.07	−2.84 *
ADHD	Sex	0.57	0.32	23.03 ***	−0.22	−2.47 *
	Age				0.07	1.62
	SES				−0.06	−2.90 **
	Working memory				0.17	2.73 **
	Regulation				0.18	2.64 **
	Inhibition				0.26	3.96 ***

**Table 5:** The analysis of the mediating effects for each dimension of EF.

	Effect Value	95% Confidence Interval		Relative Mediating Effect
		Lower Limit	Upper Limit	
Total indirect effect	−0.04	−0.07	−0.01	40.70%
Indirect effect 1 (working memory)	−0.01	−0.03	−0.00	13.95%
Indirect effect 2 (regulation)	−0.01	−0.02	0.00	7.64%
Indirect effect 3 (inhibition)	−0.02	−0.04	−0.01	19.21%
C2	0.01	−0.01	0.03	

Notes: C2 is a comparison of the difference between the indirect effect of working memory and the indirect effect of inhibition.

## Data Availability

The data presented in this study are available on request from the corresponding author.
